# Neurocognitive patterns across genetic levels in behavioral variant frontotemporal dementia: a multiple single cases study

**DOI:** 10.1186/s12883-022-02954-1

**Published:** 2022-12-06

**Authors:** Hernando Santamaría-García, Natalia Ogonowsky, Sandra Baez, Nicole Palacio, Pablo Reyes, Michael Schulte, Andrea López, Diana Matallana, Agustín Ibanez

**Affiliations:** 1 PhD program in Neuroscience, Pontificia Universidad Javeriana, Bogotá, Colombia; 2 Memory and cognition Center, Intellectus, Hospital Universitario San Ignacio, Bogotá, Colombia; 3 Department of Neurology, Global Brain Health Institute, University of California San Francisco, San Francisco, CA USA; 4 CONICET & Cognitive Neuroscience Center (CNC), Universidad de San Andrés, Buenos Aires, Argentina; 5 Faculty of Psychology, Universidad de los Andes, Bogotá, Colombia; 6 Integrated Program in Neuroscience, McGill University, Montreal, Canada; 7 Pontificia Universidad Javeriana, Bogotá, Colombia; 8 Fundación Santa Fe de Bogotá, Bogotá, Colombia; 9 Latin American Institute for Brain Health (BrainLat), Universidad Adolfo Ibanez, Santiago de Chile, Chile; 10 Cognitive Neuroscience Center (CNC), Universidad de San Andrés, & National Scientific and Technical Research Council (CONICET), Buenos Aires, Argentina; 11 Trinity Collegue of Dublin, Dublin, Irland; 12 Global Brain Health Insititute, Universidad California San Francisco-Trinity College of Dublin, San Francisco, USA; 13 Global Brain Health Insititute, Universidad California San Francisco-Trinity College of Dublin, Dublin, Irland

**Keywords:** *MAPT*, *TARDBP*, *TREM2*, *Tau* haplotypes, *APOE* variants, bvFTD, Magnetic Resonance Imaging, Clinic and neurocognitive profiles, Gene-atrophy overlap, Frontotemporal dementia, Mutations, Genetics, Structural neuroimaging, Cognition, Gene-atrophy association

## Abstract

**Background:**

Behavioral variant frontotemporal dementia (bvFTD) has been related to different genetic factors. Identifying multimodal phenotypic heterogeneity triggered by various genetic influences is critical for improving diagnosis, prognosis, and treatments. However, the specific impact of different genetic levels (mutations vs. risk variants vs. sporadic presentations) on clinical and neurocognitive phenotypes is not entirely understood, specially in patites from underrepresented regions such as Colombia.

**Methods:**

Here, in a multiple single cases study, we provide systematic comparisons regarding cognitive, neuropsychiatric, brain atrophy, and gene expression-atrophy overlap in a novel cohort of FTD patients (*n* = 42) from Colombia with different genetic levels, including patients with known genetic influences (G-FTD) such as those with genetic mutations (GR1) in particular genes (MAPT, TARDBP, and TREM2); patients with risk variants (GR2) in genes associated with FTD (tau Haplotypes H1 and H2 and APOE variants including ε2, ε3, ε4); and sporadic FTD patients (S-FTD (GR3)).

**Results:**

We found that patients from GR1 and GR2 exhibited earlier disease onset, pervasive cognitive impairments (cognitive screening, executive functioning, ToM), and increased brain atrophy (prefrontal areas, cingulated cortices, basal ganglia, and inferior temporal gyrus) than S-FTD patients (GR3). No differences in disease duration were observed across groups. Additionally, significant neuropsychiatric symptoms were observed in the GR1. The GR1 also presented more clinical and neurocognitive compromise than GR2 patients; these groups, however, did not display differences in disease onset or duration. APOE and tau patients showed more neuropsychiatric symptoms and primary atrophy in parietal and temporal cortices than GR1 patients. The gene-atrophy overlap analysis revealed atrophy in regions with specific genetic overexpression in all G-FTD patients. A differential family presentation did not explain the results.

**Conclusions:**

Our results support the existence of genetic levels affecting the clinical, neurocognitive, and, to a lesser extent, neuropsychiatric presentation of bvFTD in the present underrepresented sample. These results support tailored assessments characterization based on the parallels of genetic levels and neurocognitive profiles in bvFTD.

**Supplementary Information:**

The online version contains supplementary material available at 10.1186/s12883-022-02954-1.

## Highlights

• Different genetic levels in underrepresented FTD patients are not well understood.

• We compared cognition, neuropsychiatry, and gene-atrophy overlap in FTD cases.

• Mutations (MAPT-TARDBP-TREM2), variants (tau-APOE) & sporadic cases were compared.

• FTD genetic level was linked to clinical & neurocognitive changes in case analysis.

• Mutations showed more impairments than risk variants and sporadic presentations.

**Supplementary Information:**

The online version contains supplementary material available at 10.1186/s12883-022-02954-1.

## Background

Frontotemporal dementia (FTD) is a clinically varied neurodegenerative disease with heterogenous sporadic and genetic presentations [1–3]. The impact of these heterogeneous influences on cognitive, neuropsychiatric, and neuroanatomical phenotypes is under study. As genetic therapies are undergoing human clinical trials for genetic FTD, the characterization of genetic levels profile associated with different neuroanatomical and clinical presentations remains a critical endeavor. The sporadic (S-FTD (GR3)) vs. genetic (G-FTD) presentations exhibit different clinical and atrophy patterns [4–6]. G-FTD patients tend to be younger and present an earlier age at onset than S-FTD (GR3) [7, 8].

Different layers of genetic levels account for FTD phenotypes. In a first layer, the FTD phenotypes are generated by autosomal dominant mutations in a group of causative genes including, microtubule-associated protein *tau* (*MAPT*), progranulin (GRN), and chromosome 9 open reading frame 72 (C9orf72) causes. Each genetic group causes between ~ 5 and 10% of all FTD [9]. Rare FTD-causing mutations have also been found in the transactive response DNA binding protein of the TDP-43 gene [10]. Moreover, homozygous and heterozygous mutations in Triggering Receptor Expressed on Myeloid cells 2 (*TREM2*) may resemble FTD clinical phenotype without any bone-associated symptoms [11, 12].

Second, aside from mutations in causative genes, the FTD phenotypes have been associated with another layer of genetic levels that include allele variants in candidate genes. Although the genetics of non-monogenic FTD has been less studied, some groups have examined the potential association of FTD with the locus of *tau* haplotypes H1 and H2 of [13], and with risk variants of the apolipoprotein E (*APOE*) gene [14, 15]. Finally, different studies have reported a high prevalence of sporadic FTD phenotypes. About 60% of patients with frontotemporal dementia have no family history of dementia and are considered sporadic cases [16, 17]. Although the relation of genetic levels and bvFTD’s neurocognitive heterogeneity is a critical issue, most of previous studies comparing different profiles of G-FTD vs. S-FTD (GR3) have followed descriptive approaches. Systematic comparisons of the different genetic levels and related neurocognitive (atrophy and cognition) and neuropsychiatric profiles are still required. Moreover, most previous results did not include Latin American samples—a significantly underrepresented population with relevant genetic levels [3, 18, 19]. Moreover, no previous report has assessed the degree of gene-atrophy overlap of FTD patients in different levels. Here, in a multiple single cases study, we aimed to compare cognitive, neuropsychiatric, and brain atrophy patterns in FTD patients (*N* = 42) with different genetic levels (G-FTD), including a) GR1: patients with mutations in different types of causative genes (*MAPT*, *TARDBP*, and *TREM2*), b) GR2: patients with variants in candidate genes potentially associated with FTD (*tau* Haplotypes H1 and H2 and variants of *APOE* including *ε2, ε3, ε4*), and c) S-FTD (GR3): patients with sporadic FTD. We also investigated the gene-atrophy overlap in patients with different layers of genetic levels using previous repositories of brain regions for specific gene expression (Allen Human Brain Atlas [20, 21]). G-FTD patients vs. S-FTD (GR3) were compared following a multiple single-case approach [22–24]. This procedure allows the comparison of multiple individuals' test scores with values derived from small samples.

Considering previous evidence, we predicted a direct relationship between neurocognitive impairment and genetic levels (the greater the genetic levels, the more severe the neurocognitive impairment). Thus, increased incidence and severity of cognitive impairment (regarding executive functioning and social cognition), major neuropsychiatric disturbances, and specific frontotemporal atrophy in G-FTD compared with S-FTD (GR3) (GR1 > GR2 > GR3) was expected. Furthermore, considering the cognitive deficits associated with frontal structures, we anticipated high gene-atrophy overlap in frontal areas in G-FTD patients with GR1 in comparison to GR2. Still, neurocognitive patterns associated with different genetic levels in FTD cases in Latin American populations are not completely understood; their detailed characterization could provide important insight into the heterogeneity of the disease and may help to further define FTD phenotypes.

## Methods

### Participants

We assessed a total of forty-two FTD patients who fulfilled the revised criteria for probable bvFTD [25]. A group of neurologists, psychiatrists, neuropsychologists, and geriatricians who comprise the staff of the memory clinic at the Center for Memory and Cognition "Intellectus" at the Hospital San Ignacio in Bogotá, Colombia evaluated all patients. After this clinical assessment, a blood sample and cerebral magnetic resonance imaging (MRI) were extracted to assess each patient’s genetic characterization. Patients were selected from an ongoing protocol in Bogotá, Colombia [26–28]. Patients presented with notable changes in personality and social behavior as verified by caregivers. From this sample, we identified patients with different genetic levels including three GR1 patients with mutations in *MAPT*, *TARDBP*, and *TREM2* genes, as well as GR2 patients who exhibited different risk *tau* haplotypes (six patients) and risk variants of *APOE* (twenty-five patients).

All patients underwent a standard examination battery, including neurological, neuropsychiatric, and neuropsychological assessments, and were assessed with MRI. We also assessed a group of ten healthy controls matched to G-FTD and S-FTD (GR3) by sex, age, and years of education recruited from a larger pool of volunteers who did not have a history of drug abuse or a family history of neurodegenerative or psychiatric disorders. This group of controls was included to assess the brain atrophy pattern of G-FTD and S-FTD (GR3) patients. All participants provided written informed consent in compliance with the Declaration of Helsinki. The Ethics Committee of the Pontificia Universidad Javeriana at Bogotá Colombia approved the study (14/2021).

### Genetic assessment

Following previous procedures [29–32], we performed an extensive genetic assessment including targeted sequencing (> 300 RefSeq genes associated with neurodegenerative disorders), screening for known or novel pathogenic variants of the main dementia genes (coding and exon–intron boundary regions of GRN, *MAPT*, *TARDBP*, FUS, APP, PSEN1, PSEN2), C9orf72 repeat, dementia risk alleles including *MAPT* mutation (A152T), *MAPT* rs1052553 (which tags and discriminates between the H1 and H2 haplotypes (17q haplotype)), as well as *APOE* alleles and polymorphisms (rs429358 and rs7412). Genetic analyses discarded the presence of mutations on the next major causal genes, including GRN; C9ORF72, Presenilin 1 and 2 (PSEN1, PSEN2), and Amiloyd precursor protein (APP).

#### FTD groups

##### A) G-FTD GR1

From the total sample, analyses revealed that one patient (2.4% of the total sample) carried a missense variant of *MAPT* (c.454G > A, p.Ala152Thr, rs143624519), one patient (2.4% of the sample) had a missense variant in the *TARDBP* gene (c.1147A > G, p.I383V, rs80356740), and one patient (2.4% of the sample) exhibited a missense variant of *TREM2* (c.140G > A, p.Arg47His, rs75932628). Patients who integrated this group encompasses a patient with a mutation in one of the most described causative genes of FTD named the Microtubule-associated protein *tau* (*MAPT*) gene [33]. The *MAPT* gene involves the deposition of the hyperphosphorylated protein *tau*; it exhibits a prevalence of ∼10–20% in familial FTD cases [32, 34–36], early onset of symptoms and presents symmetrical ventral frontal and temporal brain atrophy (with other regions being less consistently reported) [37–40]. In this group, we also included a patient with a mutation in a rare causative gene, the transactive response DNA binding protein of the TDP-43 gene (*TARDBP*) [41], which account for less than 1% of familial FTD [42] and 3% of familial Amyotrophic Lateral Sclerosis (ALS) [43] cases. TARDBP mutations present with frontotemporal atrophy are associated with behavioral disturbances, including disinhibition [39, 44]. Presentations with semantic variant primary progressive aphasia have been also reported [45]. Finally, in this group, we also included one patient with a mutation in the gene encoding the Triggering Receptor Expressed on Myeloid cells 2 (*TREM2*), a significant regulator of neuroinflammatory processes in neurodegeneration [11, 12, 46], that are also associated with familial forms of FTD. A group of studies has linked *TREM2* homozygous and heterozygous mutations to FTD and suggested a pattern of frontotemporal atrophy related to behavioral and memory deficits [11, 12, 47].

##### B) G-FTD GR2

Six patients (14.2% of the sample) had *H1H2* and *H2H2 MAPT* genotype. Among these patients, three patients presented with the *H2H2* and three patients presented with the *H1H2* genotype. Among the *APOE* variants, twenty-five (59.52%) exhibited *APOE* haplotypes possibly associated with an increased risk for developing neurodegenerative disease or earlier age of onset^32,47^ (including ε2 and ε4 haplotypes). In total, eight patients carried an *APOE ε2ε3* genotype, fifteen patients exhibited the ε3ε4 genotype, and two patients had the ε4ε4 variant. The *H1 tau* haplotype is typically implicated in sporadic tauopathies and late-onset Alzheimer's disease (AD). The *H2 tau* haplotype has been more frequently associated with familial FTD [48, 49], reduced frontotemporal metabolism, and increased behavioral disturbances [50]. Similarly, FTD phenotypes have been found in carriers of genes encoding apolipoprotein E (*APOE*), specifically *APOE ε3* and *ε4* haplotypes [51, 52]. The *APOE ε3/ε4/* haplotypes may increase severity of PPA and bvFTD [15, 53]. The role of the *APOE ε2* allele remains under debate with conflicting reports of protection and increased risk [52, 54]. The bvFTD *ε4* carriers exhibited more significant atrophy in the frontal cortex, anterior insula, and cingulate cortex with a right predominance [55].

##### C) S-FTD (GR3)

We included ten patients with sporadic presentation who did not exhibit particular mutations and showed a combination of *H1H1 tau* genotype and ε3ε3 variants of *APOE*, considered as lower risk genotypes.

### Multiple single-case procedures

We used Crawford's test (a modified one-tailed t-test) [22, 23, 56–58] to compare performance in cognitive, neuropsychiatric measures and brain atrophy patterns between particular cases in the G-FTD and S-FTD (GR3) groups. Indeed, cognitive, neuropsychiatric, and brain atrophy measurements for each single-case were compared with a group of six paired S-FTD (GR3) patients counterbalanced in terms of gender, age and education factors. Through this protocol, we can compare several individuals’ scores with the norms extracted from the small sample and determine the significance of mentioned comparison. This test has a low type I error, is robust in distributions that do not satisfy normality, and has already been used in single case studies [59–61]. Only values with *p* < 0.05 were considered statistically significant. Effect sizes obtained through the same methods are reported as point estimates (zCC as effect size for the modified t-test with covariate analysis) [62].

We followed the single-case methodology to assess the cognitive and neuropsychiatric profile and the brain atrophy pattern of G-FTD patients with mutations, comparing them to the S-FTD (GR3). To perform further analyses, patients with different *tau* and *APOE* genotypes were classified into groups according to the type of risk variant. Regarding *MAPT*, three groups were created (*H1H1*, *H1H2*, and *H2H2* genotype). In the same line, we grouped patients into four groups, according to the *APOE* risk variants (*ε2ε2, ε2ε3, ε3ε4*, and *ε4ε4* genotypes). Patients of the G-FTD and S-FTD (GR3) were in the early/mild stages of the disease and did not meet the criteria for other psychiatric disorders.

### Cognitive profile

The assessment protocol included multiple instruments addressing cognitive status, executive functions, social cognition, and neuropsychiatric symptoms.

#### Cognitive screening

General cognitive state was assessed with a validated Spanish version of the Montreal Cognitive Assessment (MOCA) [63]. It includes an assessment of working memory, short-term memory, attention, language, orientation, as well as visuospatial and executive skills (alternation, phonemic fluency, and abstraction). In the MOCA, the highest score is 30 points, whereas a score of 25 or below indicates impairment.

#### Visual-constructional skills

We used the Rey–Osterrieth Complex Figure Test (RCFT) [64, 65]. This is a validated instrument used to assess visuospatial construction and visual memory abilities. Performance on this test is not directly affected by education level. The RCFT scoring system divides the complex figure into 18 units. Afterward, each unit is scored separately for accuracy and placement. Each branch of the figure receives a score of 0, 0.5, 1, or 2, and the scores are then summed to obtain the raw total score for that drawing. Therefore, the raw scores will range from 0 to 36.

#### Executive functioning

Executive functions were assessed through the INECO Frontal Screening (IFS) battery, which has been considered a sensitive neurodegenerative disease assessment tool for executive dysfunction [66–68]. Out of a maximum score of 30 points, a 25-point cut-off has shown a specificity of 91.5% and a sensitivity of 96.2% for detecting patients with dementia, which frequently suffer from dysexecutive symptoms [67]. This test is composed of the following eight tasks: (i) a motor programming test (including a Luria series of fist—edge—palm); (ii) a task of conflicting instructions (e.g., hitting the table twice when the administrator hits it only once or, on the contrary, hitting the table once when the administrator hits it twice); (iii) a test for motor inhibitory control; (iv) a task that measures numerical working memory through backward digit spans; (v) a test of verbal working memory by stating months in reverse sequence; (vi) a task of spatial working memory which consists of the modified Corsi tapping test; (vii) an evaluation of the capacity to execute abstractions by inferring the meaning of common proverbs; and (viii) a task of verbal inhibitory control which is the modified Hayling test.

#### Verbal Inhibitory control

We assessed inhibitory control using the extended version of the Hayling test [69] which measures verbal inhibitory control and can assess verbal disinhibition in patients with neurodegeneration [27, 70].

#### Social cognition task (Theory of mind, ToM)

We employed the Reading the Mind in the Eyes Test (RMET) [71] to evaluate the emotional component of the theory of mind (ToM). In Latin American patients with neurodegeneration, the RMET has previously been used to assess social cognitive functioning [27, 70]. In fact, the RMET is a validated computerized test in which 36 images are presented. Each picture depicts the face region starting from the midpoint of the nose to just above the eyebrows. Then, the participant has to choose which of the four words presented would best describe what the person in the picture may be feeling or thinking.

### Neuropsychiatric symptoms

#### Frontal Systems behavioral scale (FrSBe)

Neuropsychiatric symptoms were assessed using the FrSBe which is composed of three subfactors measuring changes in apathy, disinhibition, and dysexecutive behaviors (hereafter, referred to as disorganized behavior). Furthermore, in the FrSBe [72], the neuropsychiatric symptoms are tracked in order to determine if they were chronic (the symptoms were present before consultation) or current. This test is considered to be sensitive for monitoring behavioral changes in patients with neuropsychiatric diseases [26].

### Structural brain measures

#### Imaging recordings

All participants were scanned in a Philips Achieva 3 T scanner that had a 16-channel SENSE antenna. The anatomical and 3D T1-weighted images had the following parameters: echo time = 3.8 ms, repetition time = 7.9 ms, voxel size = 0.5 × 0.5 × 0.5 mm, 310 sections, ACQ matrix = 220 × 220 pixels.

#### Data analysis of neuroimaging data. Voxel-based morphometry (VBM) analysis

Images were preprocessed using the DARTEL Toolbox following previously described procedures [73]. Then, modulated 12-mm full-width half-maximum kernel-smoothed [74] images were normalized to the MNI space. Afterward, they were analyzed through general linear models for second level analysis using SPM-8 software (http://www.fil.ion.ucl.ac.uk/spm/software/spm8). To explore regional gray matter (GM) reduction in the G-FTD cases relative to S-FTD (GR3) patients, we performed two-sample tests including total intracranial volume as a confounding covariate (*p* < 0.001, uncorrected, extent threshold = 50 voxels).

#### Gene expression and atrophy pattern

We analyzed the potential link between each atrophy pattern in the G-FTD group and the levels of gene expression in different brain areas. To this end, we first determined the brain atrophy pattern of patients in the G-FTD group comparing each patient with a control group of ten healthy controls matched by age, sex, and education (mean age = 62.81 years, SD = 6.1; mean years of formal education = 10.61, SD = 3.7). Matching criteria for both groups were sex, age (± 5 years), and years of education (± 5 years). Then, we analyzed the overlap between brain atrophy areas in G-FTD patients and the regions with the highest expression of the genes of interest, as reported with the microarray data of the Allen Human Brain database [20, 21]. We selected probes whose signal was high above the background noise. We also established the location of the highest levels of gene expression (in MNI coordinates) in the brain of a healthy donor with similar demographic characteristics (H0351.1009). As in previous reports [61, 75], five-mm radius spherical ROIs were constructed with each coordinate to create the gene expression map. As the gene expression pattern is widely distributed across the brain and Allen Atlas presents more than 360 areas of expression of each gene, we only include the areas of significant expression. Thus, to assess the overlap between the pattern of the patient’s brain atrophy and the regions of gene expression of MAPT (probe CUST_449_PI416408490), TARDBP (probe A_23_P403955), TREM2 (probe A_23_P167941) and APOE (probe A_24_P788772), we ranked the regions by levels of expression and selected the group of brain areas positioned above the 90th percentile (12–15 brain areas, approximately). To explain that we pursue this approach following previous studies.We reported brain areas in which we found an overlap of at least 50 voxels (Figs. 5 and 6).

## Results

### Clinical, cognitive, and neuropsychiatric results

This section compares cases with different degrees of identified genetic levels (G-FTD, GR1, and GR2) and S-FTD (GR3) patients. Demographic, cognitive, neuropsychiatric, and clinical results are presented in Table 1.

**Table 1:** Demographic, cognitive, neuropsychiatric and clinical information for patients with different genetic risks

	**Cognitive and Clinical domains**	**GR1 (MAPT gene)**	**GR1 (TARDBP gene)**	**GR1 (TREM2 gene)**	**GR2 Tau Haplotypes**	**GR2 APOE Haplotypes**	**S-FTD(GR3)**
**Demographics and clinical information**	Age [mean (SD)]	66	48	63	66,99 (5,71)	70,01 (8,76)	68,29 (9,22)
	Sex (F:M)	0:01	0:01	1:00	6:06	6:06	5:05
	Educational level (years)	20	18	16	13,09 (5,29)	14,04 (6,30)	14,25 (2,38)
	Age of Disease onset	58	46	50	58,93 (7,39)	58,89 (8,10)	60,33 (10,52)
	Disease duration	8	2	13	8,88 (1,45)	9,39 (2,43)	9,87 (4,85)
**Cognitive assessment**	Moca	15	14	12	15,17 (7,83)	17,73 (6,52)	18,53 (8,24)
	Rey-Osterrieth Figure	22	33	19	23,11 (11,08)	22,07 (10,59)	29,87 (9,73)
**Executive functioning**	Ineco Frontal Screening	12	12	9	14,28 (6,61)	14,68 (7,51)	18,83 (6,29)
	Verbal inhibitory task	19	22	18	23,33 (6,65)	22,77 (12,71)	16,25 (8,46)
**Social Cognition**	Reading Mind in the eyes	9	8	9	11,41 (2,29)	11,34 (2,73)	12,12 (1,12)
	Reading Mind in the faces	9	9	8	10,44 (1,91)	12,89 (1,99)	11,87 (1,24)
**Neuropsychiatric Symptoms**	Chronic apathy	16	18	21	32,16 (3,53)	21,59 (6,08)	23,12 (9,86)
	Chronic disinhibition	18	19	19	28,66 (5,75)	21,52 (5,45)	20,62 (5,45)
	Chronic disorganized behavior	28	18	24	37,01 (11,83)	28,70 (7,79)	29,87 (7,79)
	Total Chronic Symptoms	62	55	72	76,82 (14,82)	71,61 (11,92)	73,62 (11,38)
	Current apathy	38	49	48	40,05 (9,85)	38,11 (10,75)	41,87 (10,34)
	Current disinhibition	31	37	39	30,75 (9,35)	30,40 (10,97)	26,98 (8,99)
	Current disorganized behavior	47	47	51	55,33 (10,77)	48,93 (14,06)	44,25 (11,06)
	Total Current Symptoms	116	133	138	125,66 (11,64)	117,65 (12,89)	118,51 (10,49)

### GR1: p.Ala152Thr variant (*MAPT*) vs. S-FTD (GR3)

**Fig. 1:**
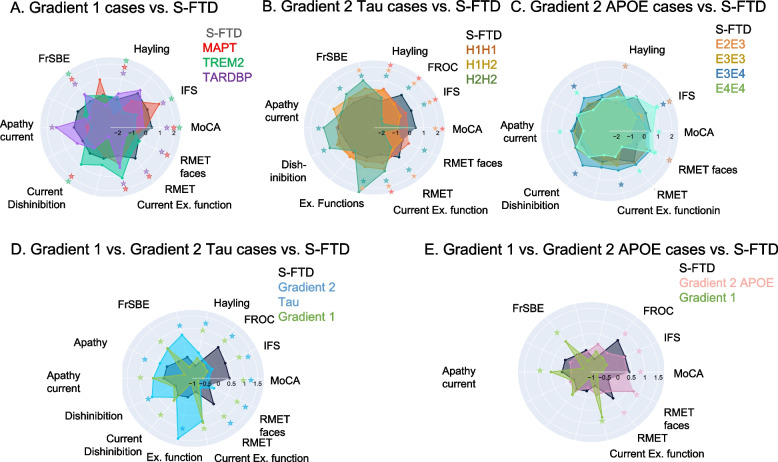
Radial Charts of cognitive and neuropsychiatric results between patients with different genetic levels groups GR1 and GR2 compared to S-FTD (GR3) patients. Each panel shows z-scores of performances of each genetic case in different cognitive and neuropsychiatric measures. Panel **A** compares z-scores of each GR1 case vs. S-FTD (GR3); panel **B** depicts z-score comparisons between GR2 *tau* haplotypes cases vs. S-FTD (GR3) patients; panel **C** shows z-score comparisons between GR2 *APOE* risky variants vs. S-FTD (GR3). Stars depict significant differences between each type of patients vs. S-FTD (GR3) (*p* < .05). Color of star coincides with color indexing each patient group/variant. Panels **D** and **E** show the comparison between z-scores of cognitive and neuropsychiatric measures between GR1 patients vs. GR2 patients with *tau* haplotypes and S-FTD (GR3) (Panel **D**), and between GR1 patients vs. GR2 patients with *APOE* variants and S-FTD (GR3) (Panel E)

The *MAPT* case showed significantly lower scores than S-FTD (GR3) patients in cognitive screening (t = -3.91, *p* < 0.01, zCC = -3.06), visual-constructional skills (t = -3.20, *p* < 0.01, zCC = -2.99), executive functioning (t = -3,89; *p* < 0.01, zCC = -3.05), and ToM in comparison to S-FTD (GR3) (t = -3.12, *p* < 0.05, zCC = -2.57). Moreover, fewer chronic neuropsychiatric symptoms than S-FTD (GR3) were revealed by lower total scores of FrSBe (t = 2.29, *p* < 0.05, zCC = 2.11), as well as disinhibition (factor: t = 2.18, *p* < 0.05, zCC = 2.14) and disorganized behavior (t = 5.78, *p* < 0.001, zCC = 5.22). No other measures reached significant values (Tables 1 and 2 and Fig. 1A). No familial antecedents of neurodegeneration were reported for this case.

**Table 2:** Differences between GR1 and GR2 patients and S-FTD (GR3) in cognitive and neuropsychiatric domains

	**Cognitive and Clinical domains**	**MAPT gene vs. S-FTD(GR3)**	**TARDBP gene vs. S-FTD(GR3)**	**TREM2 gene vs. S-FTD(GR3)**	**GR2 Tau Haplotypes vs. S-FTD(GR3)**	**GR2 APOE Haplotypes vs. S-FTD(GR3)**
**Demographics and clinical information**	Age [mean (SD)]	n.s	*P* < 0.01	*P* < 0.01	n.s	n.s
	Sex (F:M)	n.s	n.s	n.s	n.s	n.s
	Educational level (years)	n.s	n.s	n.s	n.s	n.s
	Age of Disease onset	n.s	*P* < 0.01	*P* < 0.01	n.s	n.s
	Disease duration (years)	n.s	*P* < 0.01	*P* < 0.01	n.s	n.s
**Cognitive assessment**	Moca	*P* < 0.01	*P* < 0.001	*P* < 0.05	*P* < 0.05	n.s
	Rey-Osterrieth Figure	*P* < 0.01	*P* < 0.001	n.s	*P* < 0.01	n.s
**Executive functioning**	Ineco Frontal Screening	*P* < 0.01	*P* < 0.001	*P* < 0.05	*P* < 0.001	*P* < 0.05
	Verbal inhibitory task	*P* < 0.05	*P* < 0.001	*P* < 0.05	*P* < 0.001	n.s
**Social Cognition**	Reading Mind in the eyes	*P* < 0.05	*P* < 0.05	*P* < 0.05	*P* < 0.05	n.s
	Reading Mind in the faces	*P* < 0.05	*P* < 0.05	*P* < 0.05	*P* < 0.05	n.s
**Neuropsychiatric Symptoms**	Chronic apathy	n.s	n.s	n.s	*P* < 0.01	n.s
	Chronic disinhibition	*P* < 0.05	n.s	n.s	*P* < 0.001	n.s
	Chronic disorganized behavior	*P* < 0.05	n.s	n.s	*P* < 0.001	n.s
	Total Chronic Symptoms	*P* < 0.05	*P* < 0.05	n.s	*P* < 0.01	n.s
	Current apathy	n.s	*P* < 0.05	*P* < 0.05	n.s	n.s
	Current disinhibition	n.s	*P* < 0.05	n.s	n.s	n.s
	Current disorganized behavior	n.s	n.s	*P* < 0.05	n.s	n.s
	Total Current Symptoms	n.s	*P* < 0.05	*P* < 0.05	n.s	n.s

### GR1: p.Ile383Val variant (*TARDBP*) vs. S-FTD (GR3)

The *TARDBP* case exhibited an earlier disease onset (t = -3.66, *p* < 0.01, zCC = -2.26) and a shorter disease duration (t = -6.76, *p* < 0.01, zCC = -5.26) in comparison to the S-FTD (GR3) patients. This patient had family member with ALS. This case also displayed worse scores in cognitive screening (t = -9.67, *p* < 0.001, zCC = -7.26), executive functioning (t = -6.15, *p* < 0.001, zCC = -5.17) verbal inhibition (t = -8,94; *p* < 0.001, zCC = -6.67) and ToM (t = -2.92, *p* < 0.05, zCC = -2.11) in comparison with S-FTD (GR3) patients. Fewer scores of chronic neuropsychiatric symptoms (in comparison to S-FTD (GR3)) were revealed by FrSBe total score (t = -2.29, *p* < 0.05, zCC = 2.11), but worse total current neuropsychiatric symptoms (t = 2.88, *p* < 0.05, zCC = 2.16), current apathy (t = 3.23, *p* < 0.05, zCC = 3.22), and current disinhibition (t = 2.67, *p* < 0.05, zCC = 2.06) were observed. No other measures reached significant values (Tables 1 and 2 and Fig. 1A).

### GR1: p.Arg47His variant (*TREM2*) vs. S-FTD (GR3)

This case had early disease onset (t = -4.76, *p* < 0.01, zCC = -2.26) and a longer disease duration (t = -6.76, *p* < 0.01, zCC = -4.26) accompanied by impairments in cognitive screening (t = -2.35, *p* < 0.05, zCC = -2.08), executive functioning (t = -3.35, *p* < 0.05, zCC = -2.18), verbal inhibition (t = -3,34; *p* < 0.05, zCC = -3.37) and ToM (t = -3.15, *p* < 0.05, zCC = -1.98) compared with S-FTD (GR3). Current neuropsychiatric symptoms were more pronounced in comparison to S-FTD (GR3) as revealed by FrSBe total score (t = 3.59, *p* < 0.05, zCC = 2.36), apathy (t = 4.49, *p* < 0.01, zCC = 3.36) and disorganized behavior (t = 2.99, *p* < 0.05, zCC = 1.96). No other analyses reached significant differences (Tables 1 and 2 and Fig. 1A). No familial antecedents of neurodegeneration were reported for this case.

### GR2: *H1H2* and *H2H2* genotypes (*MAPT*) vs. S-FTD (GR3)

The patients with *H1H2* and *H2H2* genotypes (six in total) showed worse scores than the S-FTD (GR3) patients with regard to cognitive screening (t = -2.47, *p* < 0.05, zCC = -0.99), visual-constructional abilities (t = -4.11, *p* < 0.01, zCC = -1.99), executive functioning (t = -7.*73*, *p* < 0.001, zCC = -6.97), verbal inhibition (t = -5,93; *p* < 0.001, zCC = -5.52), and ToM (t = -2,98; *p* < 0.05, zCC = -2.23). Furthermore, major chronic neuropsychiatric symptoms indexed by higher total FrSBE scores (t = 4.93, *p* < 0.01, zCC = 3.23), apathy (t = 4.43, *p* < 0.01, zCC = 3.03), disinhibition (t = 6.63, *p* < 0.001, zCC = 3.34), and disorganized behavior (t = 5.23, *p* < 0.001, zCC = 3.27) were observed in comparison to S-FTD (GR3) patients. No other analyses reached significant differences (Tables 1 and 2 and Fig. 1B). One patient (*H1H2*) presented one familial antecedent (bvFTD case).

### GR2: ε2ε3, ε3ε4 and ε4ε4 Variants (*APOE*) vs. S-FTD (GR3)

In comparisons to S-FTD (GR3), these five patients displayed worse scores in executive functioning (t = -3.10, *p* < 0.05, zCC = -3.46). No other analyses reached significant differences (Tables 1 and 2 and Fig. 1C). One patient carrier of ε4ε4 risk variant had familial antecedents (AD case with age of disease onset of 67 yr.).

### Summary of cognitive and neuropsychiatric results

Overall, the G-FTD patients showed major cognitive and executive alterations than S-FTD (GR3) but were less systematically impaired in chronic neuropsychiatric symptoms (Fig. 1). Similarly, GR1 patients showed earlier disease onset without differences in disease duration, worse executive functioning, and poorer ToM, but reduced chronic neuropsychiatric symptoms when compared with GR2 patients with *tau* haplotypes. Furthermore, GR1 patients showed more current neuropsychiatric symptoms than patients with *APOE* variants (Tables 1 and 2 and Figs. 1D-E). In complementary results we compared the cognitive and neuropsychiatric functioning cases with mutations (GR1) grouped vs. patients of GR2 (S2).

### VBM results

#### Global atrophy

##### GR1: *MAPT* vs. S-FTD (GR3)

**Fig. 2:**
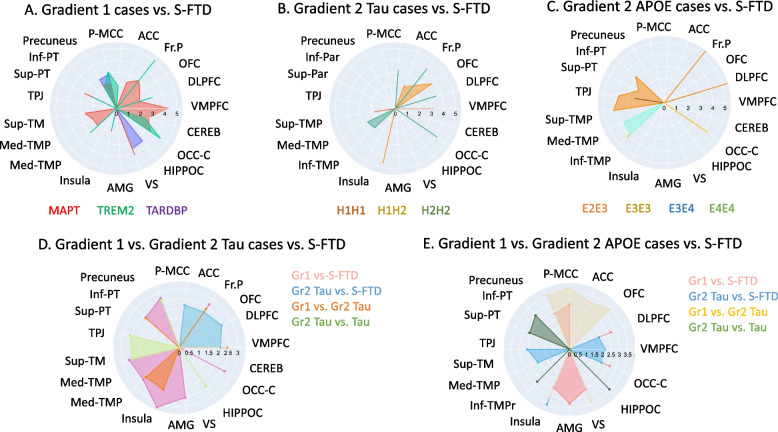
Radial Charts indexing brain atrophy between each G-FTD vs. S-FTD (GR3) patients. Each panel shows the pattern of atrophy in particular brain areas of each GR1 case vs. brain atrophy of S-FTD (GR3) patients. Panel **A** shows brain atrophy of GR1 cases, panel **B** shows brain atrophy in GR2 haplotypes of *tau* and panel **C** shows brain atrophy in GR2 patients with risk variant of *APOE*. Panel **D** and **E** show the comparison between T-scores of atrophy between the GR1 patients vs. GR2 patients with *tau* haplotypes and S-FTD (GR3) (Panel **D**), and between GR1 patients vs. GR2 patients with *APOE* variants and S-FTD (GR3) (Panel **E**)

**Fig. 3:**
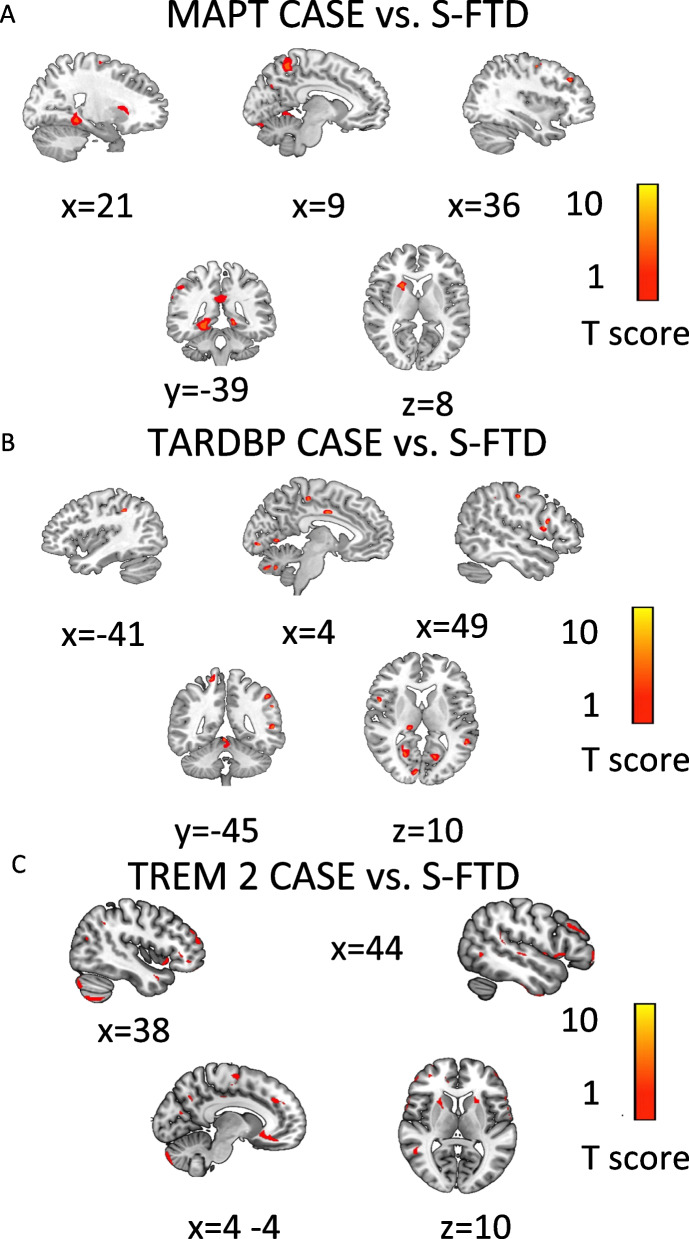
Atrophy pattern of GR1 patients compared to S-FTD (GR3). **A** Atrophy pattern of *MAPT* case compared to S-FTD (GR3). **B** Atrophy pattern of *TARDBP* case compared to S-FTD (GR3). **C** Atrophy pattern of *TREM2* case compared to S-FTD (GR3). Graph shows significant results at *p* < 0.001 uncorrected

Regarding S-FTD (GR3) patients, the *MAPT* case showed increased atrophy in the bilateral precuneus, bilateral anterior cingulate cortex, bilateral parahippocampal gyrus, middle frontal gyrus (BA46), bilateral angular gyrus, right posterior cingulate cortex (BA23), bilateral insula, and right caudate (Table 3, Figs. 2A and 3A).

**Table 3:** Brain regions (local maxima) showing significant atrophy between patients in Gradient 1 and 2 vs. S-FTD

Contrast	Region	Cluster *k*	x	y	z	Peak *t*	Peak *z*
MAPT case < S-FTD (GR3)	B Precuneus	97	6	- + 45	51	6.69	3.09
	B Anterior cingulated cortex	82	5	- + 14	42	6.56	3.70
	B Parahippocampal gyrus	95	27	- + 21	12	10.78	4.11
	L Middle frontal gyrus/BA46	94	-34	32	27	7.01	3.36
	B Angular gyrus /BA46	64	42	-60	40	10.30	4.09
	R Posterior cingulated cortex	56	6	-47	8	9.16	4.01
	B Insula	56	26	+ -23	-8	7.99	3.09
	R Caudate	109	-14	12	6	8.56	3.70
TARDBP case < S-FTD (GR3)	R Posterior cingulated cortex	79	6	-45	8	7.99	3.09
	B Anterior cingulated cortex	80	5	+ -12	40	7.56	3.70
	R Precuneus	95	6	+ -47	48	8.81	3.57
	B Parahippocampal gyrus	54	27	+ -21	12	7.01	3.36
	L Middle frontal gyrus/BA8	64	-30	36	48	8.30	3.79
	B Superior Parietal cortex	56	15	-52	66	6.16	3.65
	R cerebellum	124	24	45	-12	6.65	3.09
TREM2 case < S-FTD (GR3)	B Orbitofrontal cortex	79	24	+ -45	-12	7.89	3.09
	B Anterior cingulated cortex	80	5	+ -12	40	7.06	3.70
	R Dorsal lateral Prefrontal cortex	95	36	-12	35	7.11	3.57
	R Insula	54	27	-21	-12	6.12	3.36
	B Superior temporal cortex	64	58	-37	9	5.93	3.79
	B Cerebellum	56	24	- + 45	-12	9.16	3.65
H1H2 cases < S-FTD (GR3)	R Orbitofrontal cortex	79	24	+ -45	-12	10.09	4.09
	R Insula	80	27	-21	40	7.06	3.70
	R Medial temporal gyrus	112	40	-1	48	7.11	3.57
H2H2 cases < S-FTD (GR3)	R Dorsolateral prefrontal cortex	59	-36	12	37	6.03	3.09
	R Anterior cingulated cortex	82	5	+ -12	37	5.66	3.70
	R Superior parietal lobe	95	15	-52	66	6.78	3.57
	L Medial temporal gyrus	94	40	2	-16	7.01	3.36
APOE E2E3 cases < S-FTD (GR3)	R Medial temporal gyrus	70	40	-1	-48	7.59	3.09
	R Inferior temporal gyrus	82	-36	12	37	8.56	3.70
APOE E3E4 cases < S-FTD (GR3)	B Angular gyrus	109	24	45	-12	10.02	4.09
	R Dorsolateral prefrontal cortex	88	-36	12	37	7.86	3.70
APOE E4E4 cases < S-FTD (GR3)	R Medial temporal gyrus	66	40	-1	-48	11.01	4.09
	L Inferior parietal lobe	82	-46	-36	42	9.06	3.70
S-FTD (GR3) < GR1and GR2							
S-FTD (GR3) < MAPT case	B Medial Cingulated cortex	62	5	+ -12	37	5.06	3.09
	R Caudate	65	-14	12	6	5.11	3.27
	R Putamen	65	-17	12	9	5.26	3.45
S-FTD (GR3) < TARBP case	R Superior parietal lobe	56	15	-52	66	5.22	3.44
	B Inferior temporal lobe	52	-36	12	37	5.33	3.49
	L Anterior Cingulated cortex	58	5	+ -12	37	5.02	3.09
S-FTD (GR3) < APOE ε4	R Superior parietal lobe	56	15	-52	66	5.12	3.37
	B Anterior cingulate cortex	52	-36	12	37	5.42	3.43

*L* Left, *R* Right, *B* Bilateral

##### GR1: *TARDBP* vs. S-FTD (GR3)

Compared to S-FTD (GR3) group, the *TARDBP* case showed increased atrophy in the right posterior cingulate cortex (BA23), bilateral anterior cingulate cortex, right precuneus, bilateral parahippocampal gyrus, middle frontal gyrus (BA46), left superior parietal cortex (BA40), right cerebellum cortex, and right primary sensory cortex (Table 3, Figs. 2A and 3B).

##### GR1: *TREM2* vs. S-FTD (GR3)

Compared to S-FTD (GR3) patients, the *TREM2* risk case showed significant atrophy in the bilateral anterior cingulate cortex, bilateral orbitofrontal cortex, right dorsolateral prefrontal cortex insula, bilateral superior temporal sulcus, and cerebellum (Table 3, Figs. 2A and 3C).

##### GR2: *Tau* risk haplotypes vs. S-FTD (GR3)

**Fig. 4:**
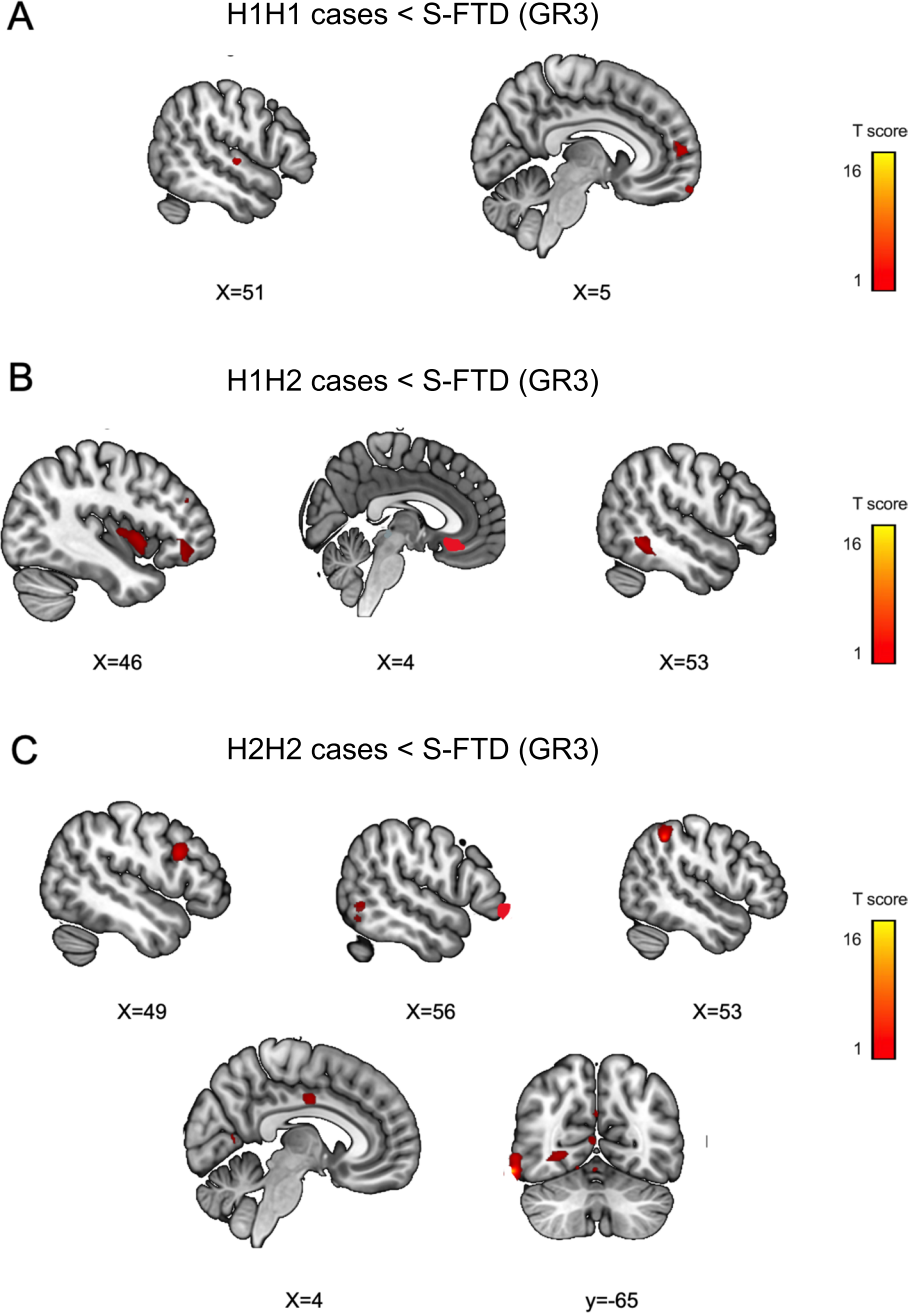
Atrophy pattern of GR2 patients with *tau* haplotypes (*H1H2*, *H2H2*) compared to S-FTD (GR3). Atrophy pattern of patients with risk haplotypes of **A** *MAPT* (*H1H2*), **B** *H1H2*, and **C** *H2H2* compared to S-FTD (GR3). Graph shows significant results at *p* < 0.001 uncorrected

Compared to S-FTD (GR3), *H1H2* haplotypes showed significant atrophy in the right insula, the right orbitofrontal cortex, and the right medial temporal gyrus. *H2H2* haplotypes exhibited significant atrophy compared to S-FTD (GR3) patients in the right dorsolateral prefrontal cortex, right anterior cingulate cortex, right superior parietal lobe, and left medial temporal gyrus (Table 3, Figs. 2B and 4A-C).

##### GR2: *APOE* risk variants vs. S-FTD (GR3)

**Fig. 5:**
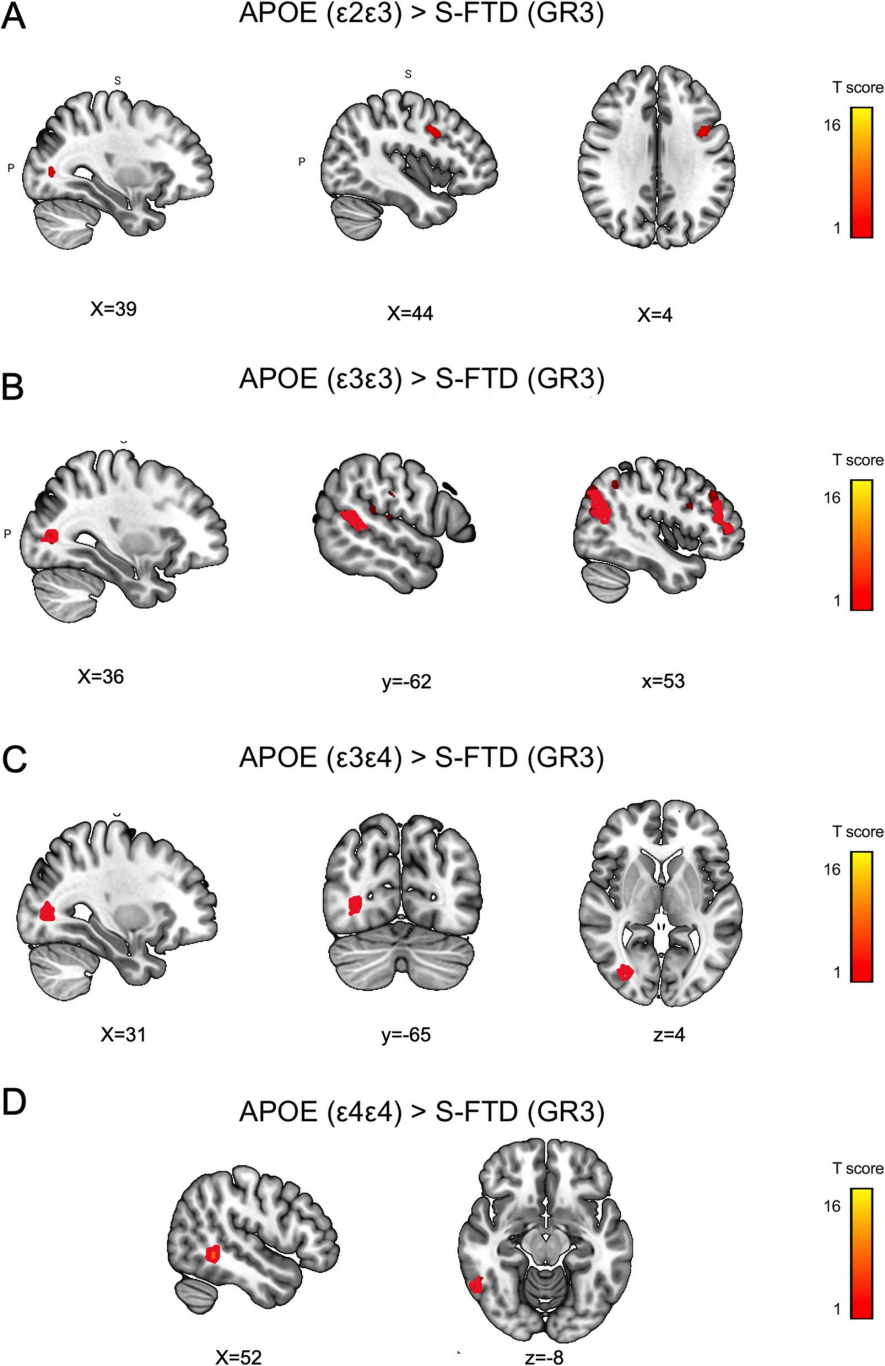
Atrophy pattern of GR2 patients with *APOE* risk variants (*ε2ε3, ε3ε3, ε3ε4, ε4ε4*) compared to S-FTD (GR3). Atrophy pattern of patients with risk haplotypes of **A** *APOE ε2 ε3*, **B** *ε3ε3*, **C** *ε3ε4*, **D** *APOE ε4* compared to S-FTD (GR3) patients. Graph shows significant results at *p* < 0.001 uncorrected

*APOE ε2ε3* haplotypes showed significant atrophy in the right medial parietal lobe, the right inferior temporal gyrus, in comparison to S-FTD (GR3). Similarly, ε3ε4 haplotypes presented more atrophy in the bilateral angular gyrus and right dorsolateral prefrontal cortex than S-FTD (GR3) patients. Patients with ε4ε4 haplotypes had increased atrophy in the right medial and inferior parietal lobe (Table 3, Figs. 2C and 5A-D).

### Global atrophy of genetic cases (G-FTD) with different genetic levels (GR1 and GR2) compared to healthy controls

#### GR1 patients

##### Patient with mutation in MAPT gene

**Fig. 6:**
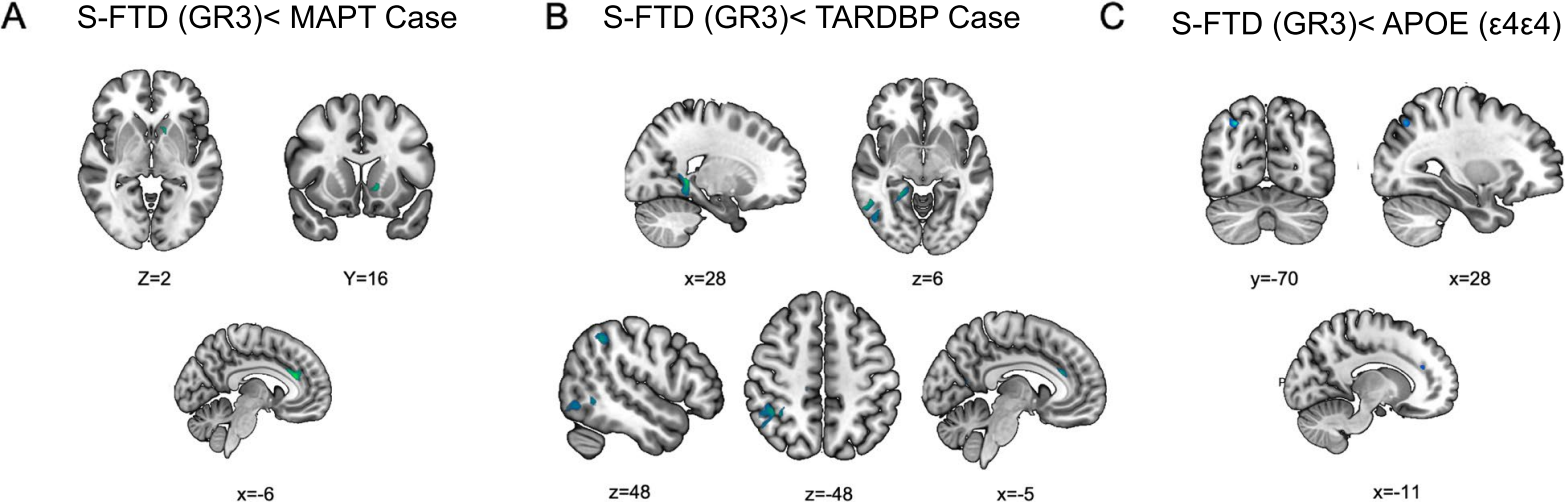
Atrophy pattern of S-FTD (GR3) < patients with different genetic levels (GR1 vs. GR2). **A** Larger atrophy pattern of S-FTD (GR3) patients vs. GR1 patient with *MAPT* mutation. **B** Brain atrophy pattern of S-FTD (GR3) patients vs. GR1 patient with *TARDBP* mutation. **C** Atrophy pattern of S-FTD (GR3) patients vs. GR2 patients with ε4ε4 variants of *APOE*. Graph shows significant results at *p* < 0.001 uncorrected

Regarding healthy controls, the patient with MAPT mutation showed extensive atrophy including GM reductions in the bilateral precuneus, bilateral parietal cortex, bilateral temporal poles, occipital areas, bilateral insula, bilateral medial cingulate cortices, right orbitofrontal cortex, medial frontal cortices, left superior temporal gyrus, parahippocampal gyrus and cerebellum cortex (Fig. 6 Panel A1).

##### Patient with mutation in TARDBP gene

Compared to healthy controls, the patient with TARDBP mutation showed GM reductions in the left medial temporal cortex, bilateral medial frontal gyrus, bilateral parietal cortex, right precuneus, right posterior cingulate cortex and right orbitofrontal cortex (Fig. 6 panel B1).

##### Patient with mutation in TREM2 gene

Compared to healthy controls, the patient with TREM2 mutation showed GM reductions in the right superior and inferior temporal cortex, bilateral medial frontal gyrus, bilateral precuneus, left medial and posterior cingulate cortex, left orbitofrontal cortex, bilateral ventral striatum, and cerebellum (Fig. 6 panel C1).

#### GR2 patients

##### Patients with H1H2 and H2H2 haplotypes of MAPT

**Fig. 7:**
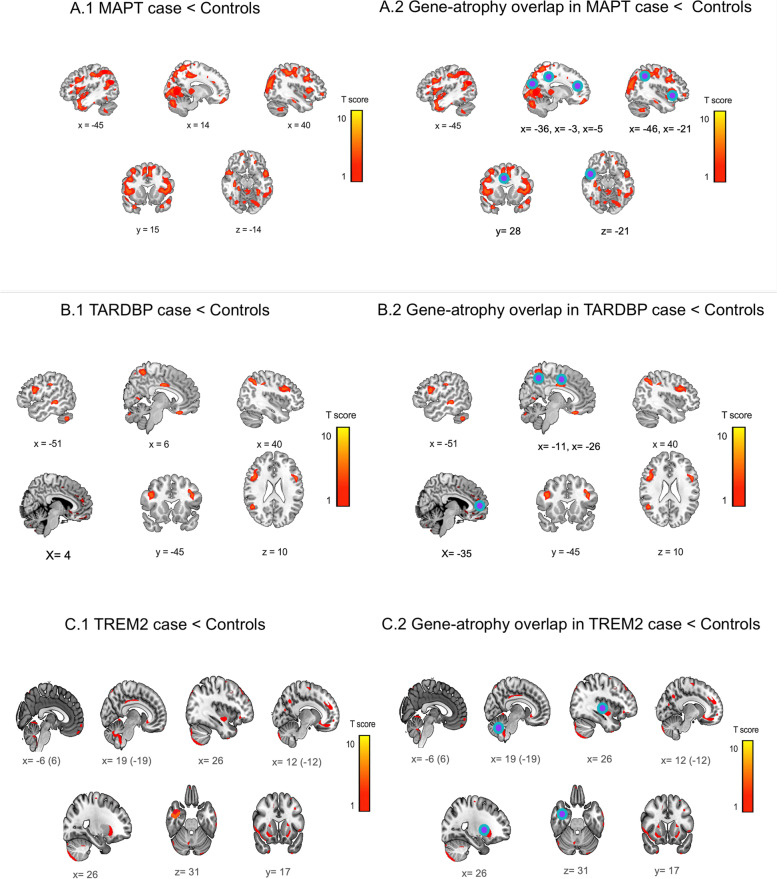
Comparisons of atrophy and gene-atrophy overlap in GR1 patients and controls. (**A**.1) Atrophy pattern in the patient with *MAPT* mutation vs. healthy controls. (**A**.2) Overlapped gene-atrophy between brain atrophy of patient with *MAPT* mutation and the expression of *MAPT* gene in the Allen Atlas. (**B**.1) Atrophy pattern in the patient with *TARDBP* mutation vs. healthy controls. (**B**.2) Overlapped gene-atrophy between brain atrophy of patient with *TARDBP* mutation and the expression of *TARDBP* gene in the Allen Atlas. (**C**.1) Atrophy pattern in the patient with *TREM2* risk variant vs. healthy controls. (**C**.2) Overlapped gene-atrophy between brain atrophy of patient with *TREM2* mutation and the expression of *TARDBP* gene in the Allen Atlas. Graph shows significant results at *p* < 0.001 uncorrected. Blue-violet dots depict peaks (more than 50 voxels) of overlap

In relation to healthy controls, the patients with H1H2 and H2H2 haplotypes of MAPT showed reduced GM in bilateral insula, bilateral anterior cingulate cortices, and bilateral precuneus, right superior temporal lobe and occipital areas (Fig. 7 panel A1).

**Fig. 8:**
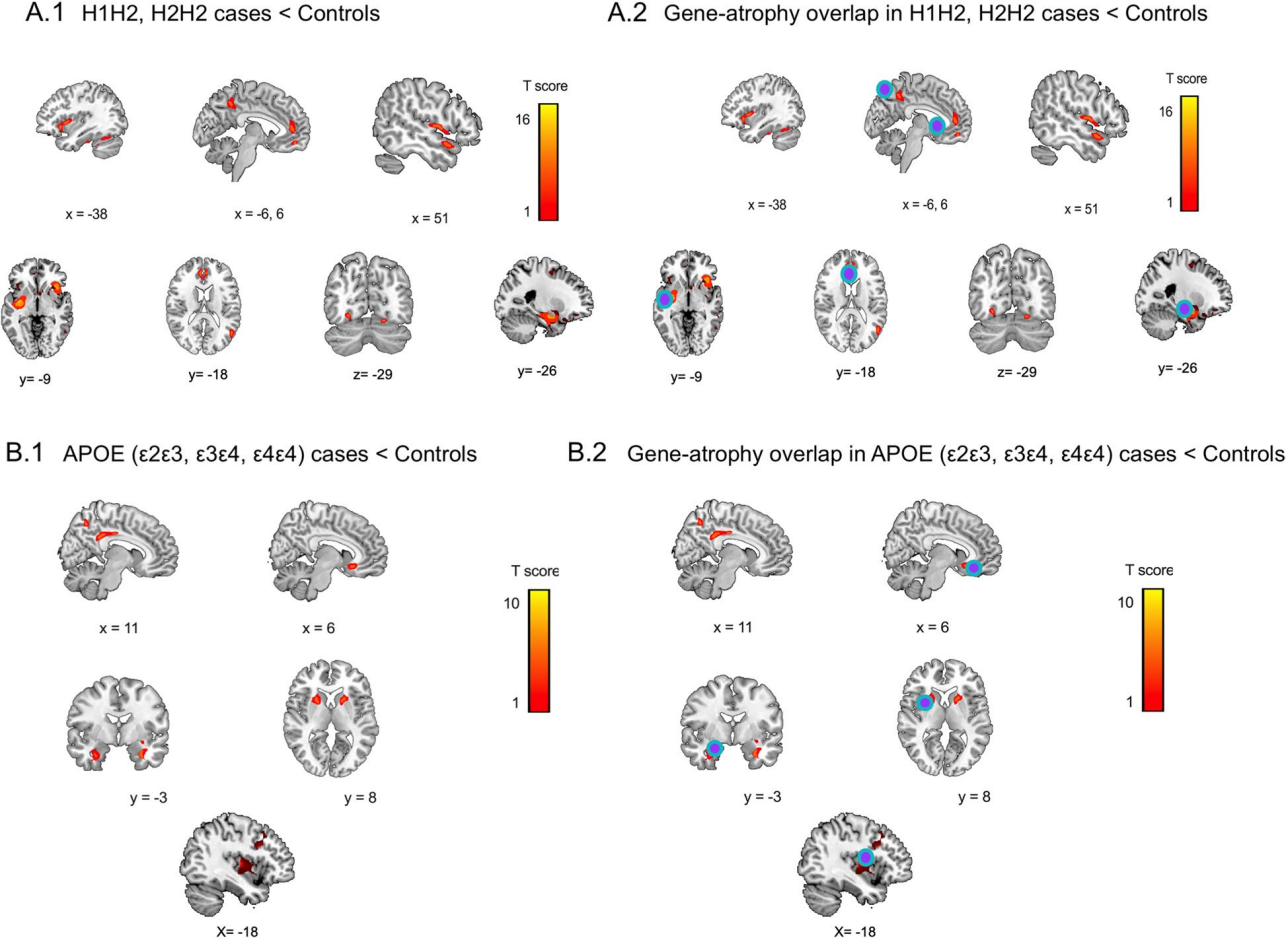
Comparisons of atrophy and gene-atrophy overlap in GR2 patients and controls. (**A**.1) Atrophy pattern in patients with H2 haplotypes vs. healthy controls. (**A**.2) Overlapped gene-atrophy between brain atrophy of patients with *H1H2*/*H2H2* variants and the expression of *MAPT* gene in the Allen Atlas. (**B**.1) Atrophy pattern in patients with specific variants of *APOE* (*ε3ε3, ε3ε4, ε4ε4*) vs. healthy controls. (**B**.2) Overlapped gene-atrophy between brain atrophy of patients with the aforementioned *APOE* variants and the expression of *APOE* gene in the Allen Atlas. The graph shows significant results at *p* < 0.001 uncorrected. Blue-violet dots depict peaks (with > 50 voxels) of overlap

##### Patients with ε2ε3, ε3ε4, ε4ε4 variants of APOE

In relation to healthy controls, these patients showed significant brain atrophy in the right posterior cingulate cortex, right precuneus, right orbitofrontal cortex, bilateral parahippocampal gyrus, and bilateral caudate (Fig. 7 panel B1).

##### Inverse patterns of brain atrophy (S-FTD > G-FTD)

We also analyzed the cases in which the S-FTD (GR3) patients exhibited greater brain atrophy than G-FTD patients. The degree of brain atrophy in the S-FTD (GR3) > G-FTD direction measured by t-peak values was significantly lower than opposite contrasts (G-FTD > S-FTD (GR3)). The S-FTD (GR3) presented more atrophy than the *MAPT* case in the bilateral medial cingulate cortex, right caudate, and right putamen (Fig. 6A). S-FTD (GR3) also showed significantly greater atrophy compared to the *TARDBP* case in the right superior parietal lobe, bilateral inferior temporal lobe, and left anterior cingulate cortex (Fig. 6B). Finally, the S-FTD (GR3) patients exhibited increased atrophy compared to patients with *APOE ε4* haplotypes in the right superior parietal lobe and bilateral anterior cingulate cortex (Fig. 6C).

##### Summary of brain atrophy results across patients with different genetic levels

In comparison to S-FTD (GR3), patients with G-FTD exhibited increased atrophy in the ventromedial prefrontal areas, frontal pole, superior temporal gyrus, ventral striatum, insula, dorsolateral prefrontal areas, orbitofrontal areas, precuneus, posterior and anterior cingulate cortices, medial temporal lobes, and angular gyrus. Furthermore, GR1 patients showed more atrophy in frontal areas, cingulated cortices, inferior temporal cortices, and precuneus than GR2 patients (*tau* haplotypes and *APOE* variants). The opposite contrast (GR2 > GR1) revealed a smaller pattern of atrophy in parietal and medial temporal areas (for a further review of coordinates and stats of areas reported see Table 3).

### Gene expression and atrophy overlap

#### GR1: *MAPT*

Five areas of atrophy overlapped with the *MAPT* gene expression in the Allen database [20, 21] (Fig. 7A.1-A.2, S2) involving the left precuneus, left posterior and anterior cingulate cortices, right angular gyrus, and right insula.

#### GR1: TARDBP

Three areas of atrophy overlapped with an expression of the *TARDBP* gene (Fig. 7B.1-B.2, S2) indexing the left precuneus, left posterior, and anterior cingulate cortices.

#### GR1: *TREM2*

Three areas of atrophy overlapped with the *TREM2* gene expression (Fig. 7C.1-C.2, S2) including the right insula, right inferior temporal lobe, left precuneus, left posterior and anterior cingulate cortices.

#### GR2: *Tau* risk haplotypes

Six areas of atrophy overlapped with the gene expression (Fig. 8 A.1-A.2, S2) including the left precuneus, left anterior cingulate cortex, right temporal lobe, right insula, and right dorsolateral prefrontal cortex.

#### GR2: *APOE* variants

Three areas of atrophy overlapped with variants (*ε3, ε4*) and *APOE* gene (Fig. 8 B1-B2, S2) in the bilateral precuneus, left anterior, and posterior cingulate cortices.

## Discussion

Here, in a multiple sinlge cases study, we assessed cognitive and neuropsychiatric profiles, brain atrophy, and gene-atrophy overlap in FTD patients with different genetic riks. G-FTD patients presented earlier disease onset, more pervasive cognitive impairments, and more atrophy than S-FTD (GR3) patients. To a lesser extent, more significant neuropsychiatric symptoms were observed in the G-FTD group (but this pattern was not consistent across GR1 patients). Across G-FTD patients, the gene-atrophy overlap analysis revealed convergent areas of atrophy in regions with specific genetic overexpression. Together, our results reveal differential genetic influences of the clinical, neurocognitive, and (to a lesser extent) neuropsychiatric bvFTD presentation.

### Results in GR1 patients

We reported a *MAPT* p.Ala152Thr variant located in a non-conserved, proline-rich, and faraway region of the repeat domain almost adjacent to the potential proline-directed phosphorylation site in PHF-*tau*. The p.Ala152Thr carriers significantly increase FTD and AD risk [40, 76]. Our case presented the more pronounced three cardinal symptoms in *MAPT* presentation [36, 77] in comparison with S-FTD (GR3) patients. Brain atrophy was extensive in fronto-insulo-parietal regions (Figs. 2 and 3A) classically affected in FTD [78–80] and in *MAPT* mutations [6, 81, 82]. The gene-atrophy overlap evidenced a spatial convergence of overexpression of *MAPT* isoforms and atrophy regions (left precuneus, left posterior and anterior cingulate cortices, right angular gyrus, and right insula; Fig. 7A).

We identified one patient with the p.Ile383Val mutation in *TARDBP* located in exon 6, a highly conserved domain involved in regulation of splicing activity of TDP-43 protein, previously reported in FTD patients [83]. Although previous studies have associated TARDBP mutations with ALS (those mutations explain near to 3% of cases), a recent study has shown that the p.Ile383Val mutation is also associated with complex FTD phenotyping, including complex proteinopathy associated in patients with semantic dementia and bvFTD [84, 85]. Our case showed early disease onset, shorter disease duration and worse scores in cognitive screening, executive functions, ToM skills, and neuropsychiatric symptoms in consonance with previous reports [39, 86]. Major atrophy involved typical regions (anterior cingulate cortex, right precuneus, middle frontal gyrus, parietal cortex, and cerebellum) [78–80] as well as parahippocampal gyrus (involved in episodic memory processing [38]), and somatosensory cortices (compromised in FTD patients with autonomic disregulation [87]). Several sites of expression of the p.Ile383Val variant presented atrophy peaks (left precuneus, left posterior cingulated cortex, and bilateral anterior cingulate cortices, Fig. 7B).

The *TREM2* patient’s mutation is associated with familial FTD [11, 12, 46] and the nonsense variant (p.Arg47His) involves immune cells and interference with anti-inflammatory function [46, 88]. In comparison to S-FTD (GR3), this case exhibited longer disease duration, more impaired cognition, and increased neuropsychiatric symptoms. These cognitive-behavioral profiles replicate *TREM2* FTD phenotypes [46, 88]. Similar to other samples from other regions [11, 46], brain atrophy revealed a fronto-inuslo-temporal compromise (anterior cingulate cortex, orbitofrontal cortex, right dorsolateral prefrontal cortex, right insula, bilateral superior temporal sulcus, and cerebellum). Multiple atrophy peaks were observed in regions of *TREM2* p.Arg47His variant expression (Fig. 7C).

### GR2: *Tau *Haplotypes and *APOE*

As expected, *tau* haplotypes (*H1H2* and *H2H2*) showed impairments in cognition (general cognitive state, visual-constructional skills, executive functioning, and ToM skills) and more severe chronic neuropsychiatric symptoms (total scores, disinhibition, apathy, and disorganized behavior) compared to S-FTD (GR3) [36, 77]. *Tau* haplotype patients exhibited more chronic neuropsychiatric symptoms than GR1 patients (Fig. 1D and Tables 1 and 2) supporting a more rapid progression of G-FTD than S-FTD (GR3) [89, 90], but also a progressive development of behavioral changes in *tau* haplotypes [38, 90–92]. Our results provide novel evidence of GR1 patients showing rapid neuropsychiatric development, with *tau* haplotypes presenting more insidious behavioral disturbances^15^. Significant atrophy in prefrontal, temporal, and basal ganglia regions were observed in risk *tau* haplotypes compared to S-FTD (GR3), although these patterns varied across *H1H1*, *H1H2*, and *H2H2* haplotypes (Table 3 and Figs. 2 and 4A-C). Six atrophy peaks overlapped with the expression of the *MAPT*, including the left precuneus, left anterior cingulate cortex, right temporal lobe, right insula, and right dorsolateral prefrontal cortex.

*APOE* risk variants exhibited worse cognitive screening, reduced executive functioning, and significantly more severe apathy compared S-FTD (GR3) patients, but fewer scores of current neuropsychiatric symptoms than GR1 patients (Fig. 1E and Tables 1 and 2). Atrophy was less typical and more posterior (parietal and inferior temporal gyrus in ε2ε3 patients, angular gyrus in ε3ε4 patients, and parietal lobe in ε4ε4 carrires, Fig. 2E). Atrophy patterns in *APOE* risk variants overlapped with the *APOE* gene expression (mostly posterior regions). These results are compatible with descriptive reports of *APOE* haplotypes, though some of its roles in FTD remain under debate [15, 51, 52, 93].

### Genetic levels parallel neurocognitive patterns

Together, our results reveal that particular genetic levels differentially compromise the neurocognitive, clinical, and, to a lesser extent, neuropsychiatric presentation of bvFTD (GR1 > GR2 > GR3) in this Colombian cohort. In comparison to patients with risk *tau* haplotypes and risk *APOE* variants, each one of the patients in GR1 (*MAPT* + *TARDBP* + *TREM2* cases) had an earlier disease onset (without differences in disease duration), increased cognitive deficits, and a more minor presence of chronic symptoms. Furthermore, all GR1 patients showed increased atrophy compared to GR2 and GR3 in classical FTD brain regions. Our results confirm a suggested major neurocognitive compromise in GR1 [47, 94] and a more chronic progression of neurocognitive and behavioral impairments in GR2 patients than in GR1 [15, 48, 52, 90, 92]. This report suggests a novel hierarchical, multimodal (behavior, cognition, atrophy, brain-gene overlaps) genetic levels across the FTD presentations (GR1 > GR2 > GR3).

Fewer FTD-relevant areas presented an inversed atrophy pattern (GR2 > GR1) in parieto-temporal regions, similar to other reports [5, 6]. Similarly, increased temporal atrophy pattern was observed in patients with *tau* haplotypes [7, 36] and *APOE* risk variants [39] when compared with S-FTD (GR3) patients. Similarly, a small increase in atrophy was observed in the S-FTD (GR3) compared to GR1 (*MAPT*: cingulate, caudate, putamen; *TARDBP*: parietal and temporal lobe, anterior cingulate cortex) and GR2 patients (*APOE ε4*: superior parietal lobe and anterior cingulate cortex). Although there is no specific explanation for this atrophy pattern and the degree of brain atrophy of these inverted patterns was smaller, expression of undetermined gene risk variants on brain tissue and differential compensatory and/or plastic effects [39, 90] would explain this pattern. More research is required to elucidate this issue.

As hypothesized, patients with higher genetic levels (GR1, specific mutations) exhibited significant frontal and temporal atrophy associated with earlier and greater cognitive impairments than GR2 patients (carriers of risk *tau* and *APOE* variants) and patients with sporadic forms of the disease (GR3). Results suggest that being a carrier of mutations in *MAPT*, *TARDBP*, and *TREM2* triggers and accelerates the development of FTD proteinopathies that, eventually, yield a more pronounced effect on the the cognitive, behavioral, and brain tissue impacts associated with FTD. Although present results support previous studies [42, 47, 95, 96], we found novel evidence regarding a significant neurodegenerative progression of FTD in cases with mutations, followed by patients with risk *tau* haplotypes or *APOE* variants, and finally sporadic FTD presentations.

Despite being a multiple single-case studies, our results align with recent studies evaluating associations between genetics, brain volume, and cognition in FTD. Present results coincided with previous findings revealing heterogeneous patterns of gray matter in symptomatic MAPT cases, but with predominant compromise of hippocampal, parahippocampal, temporal, and anterior cingulated cortices, as well as insula being associated with reduced memory and impaired executive functioning [36, 38, 47, 97]. Our results add new pieces of evidence by revealing a more expanded pattern of brain atrophy and cognitive impairments in the MAPT case extending to the precuneus and angular gyrus volume and the impairments in memory, spatial and social cognition.

Classical reports of FTD-TARDBP revealed a diffuse pattern of atrophy affecting temporal, orbitofrontal, and cingulate cortices in FTD patients associated with cognitive and semantic deficits [45, 85, 98, 99]. Our work shown an even more extended pattern of atrophy beyond classical frontal, temporal, cingulated regions, extending to the precuneus, parahippocampal cortex, cerebellum, and sensory cortex. Moreover, a global impairment in cognitive, social-cognitive functions, and increased neuropsychiatric symptoms was observed.

Regarding TREM 2 cases, our results showed significant atrophy of fronto-cingulo-temporal cortices accompanied by impaired cognitive, social-cognitive, executive, and behavioral alterations. To date, most studies have related TREM 2 mutations to AD [46, 95, 100–102], although some reports evidenced FTD phenotypes [12, 88, 103, 104]. Present results align with previous studies showing a frontotemporal brain atrophy and impaired executive function [105, 106].

The tau GR2 cases exhibited severe chronic neuropsychiatric symptoms (total scores, disinhibition, apathy, and disorganized behavior) compared to S-FTD (GR3), supporting previous studies [36, 77]. However, our results add new evidence by revealing patients with tau haplotypes displayed more insidious behavioral disturbances associated with significant atrophy in prefrontal, temporal, and basal ganglia regions.

Although the role of APOE haplotypes in FTD is still under debate, our results align with descriptive reports of *APOE* haplotypes displaying parietal and posterior atrophy associated with memory and spatial deficits [15, 51, 52, 93]. Our results add new information by revealing also increased impairments in memory, executive functioning, and apathy compared to S-FTD (GR3) patients.

Together, although present results are based on reduced sample sizes and single cases, they bring novel insights about the genotype–phenotype interactions of FTD in underrepresented populations. Results highlight differential clinical-cognitive patterns that fit with different levels of genetic burden on FTD. Results support international initiatives calling to increase the underrepresented samples to asses genetics of FTD, as recently evaluated in other reports [107, 108]. Our results add new evidence regarding genotype–phenotype interactions in FTD in LAC and particularly in Colombia. These results are relevant to address the potential ethnic factors associated with genetically mediated proteinopathies [109].

### Limitations of the multiple single-case approach

Our results should be analyzed with caution, considering the small group of patients with specific mutations in causative and candidate genes and risk variants. However, to reduce a potential statistical bias when we run comparisons, we used the Crawford index, which allows us to assess differences between single cases and control samples with statistics values [23, 24, 110]. The Crawford index has also proved statistical value in assessing brain-genetics-behavior interactions between single cases of patients with neurologic conditions in comparisons with control groups [60, 61, 111–114].

Moreover, concerning brain volume analyses, we used a lenient neuroimaging approach to the whole brain (*p* < 0.001, uncorrected, extent threshold = 50 voxels) as we followed case–control multiple single-case designs. This threshold is suggested for small studies to avoid detrimental effects of liberal primary thresholds on false positives [115] and to obtain a desirable balance between types I and II error rates [116]; it is comparable to an FDR correction of *p* = 0.05 [116]. Still, future studies with larger samples, greater statistical power, and more stringent criteria should replicate present results. Although most of the patients of this cohort of patients have been already reported [26, 27], we did not have access to cerebrospinal fluid, positron emission tomography tracking tau deposits or pathology to confirm the diagnosis. Future studies should assess the interactions between genetics, neurocognitive phenotypes, including confirmatory diagnosis biomarkers. Additional studies assessing the impacts of ancestry, admixtures, and their interaction with environmental factors should test the FTD phenotypes reported here.

A possible confound of the observed pattern of results may be disease duration. Our analyses, however, discarded this possibility as we did not find differences in disease duration between GR1, GR2, and GR3 patients. Similarly, results could be biased by differences in disease detection in patients with familial antecedents (i.e., it could be more likely to detect early symptoms in cases with previous relative disease presentations). Nevertheless, no differences in familial antecedents were observed in GR1 patients. Moreover, only two patients (out of twelve) in GR2 presented familial antecedents. Together, both patterns (disease duration and family antecedents) seem to not bias the differences across groups. Future confirmation with larger samples, however, will allow for a more systematic control of these factors.

Finally, regarding behavioral alterations, we assessed typical behavioral disturbances in FTD patients, including apathy, disinhibition, and executive problems, using a single assessment, the FrSBe [117]. This instrument has been associated to brain atrophy [26], cognitive impairments [118] and it is useful to discriminate FTD from other neurodegenerative conditions [26, 72, 119]. However, another potential limitation of our work is the absence of additional information on other possible neuropsychiatric symptoms, including depression, anxiety, psychosis, or sleep problems [120]. Future studies should track the associations between genetics and neurocognitive patterns with others behavioral alterations, including using standardized instruments such as the Neuropsychiatric Inventory (NPI) [121] or the Mild behavioral Inventory-C [122].

## Conclusions

Results suggest different neurocognitive and neuropsychiatric profiles in FTD patients dependening on the genetic level. A more severe neurocognitive compromise was observed in patients with particular mutations in risk genes than in patients with risk *tau* and *APOE* variants and S-FTD (GR3). Findings highlight the need for more differentiated assessments and interventions according to the neurogenetic and cognitive profiles of frontotemporal dementia.

## Supplementary Information

**Additional file 1. **

## Data Availability

The data are not publicly available due to restrictions because it contains information that could compromise the privacy of research participants. Data are however available from the corresponding author upon reasonable request and with permission of patients, caregivers and Hospital Universitario San Ignacio.

## Abreviations

bvFTD: Behavioral variant of Frontotemporal dementia
ALS: Amyotrophic Lateral Sclerosis
PPA: Primary progressive aphasia
*MAPT gene*: Microtubule-associated protein *tau* gene
*TARDBP gene*: The transactive response DNA binding protein of the TDP-43 gene
*TREM 2 gene*: Triggering Receptor Expressed on Myeloid cells 2 gene
APOE: Apolipoprotein E
GRN gene: Granulin precursor gene
FUS gene: Fused in sarcoma gene
C9orf72 gene: chromosome 9 open reading frame 72
PSEN 1: Presenilin 1 gene
PSEN 2: Presenilin 2 gene
G-FTD: Patients with frontotemporal dementia due to known genetic factors
GR1: Patients with genetic mutations associated with frontotemporal dementia
GR2: Patients with risk variants in candidates genes associated with frontotemporal dementia
GR3: Patients with frontotemporal dementia without known genetic sources
S-FTD: Patients with sporadic-forms of frontotemporal dementia
MRI: Magnetic Resonance Imaging
VBM: Voxel Based Morphometry
