# Supplementary material for: Neurocognitive patterns across genetic levels in behavioral variant frontotemporal dementia: a multiple single cases study
Source: BMC Neurol. 2022 Dec 6;22:454. doi: 10.1186/s12883-022-02954-1 (PMC9724347; doi:10.1186/s12883-022-02954-1)
Supplement: Supplementary file 1 — Additional file 1. [file 12883_2022_2954_MOESM1_ESM.docx]

**Supplementary Information**

**S1. Genetic Screening protocol and methods**

**Targeted sequencing.** Over 300 RefSeq genes related to neurodegenerative disorders were screened. At the UCLA Neuroscience Genomics Core, a custom-designed library was employed for exons (SeqCap EZ Choice Library, NimbleGen) and the sequencing was done on an Illumina HiSeq4000 (www.semel.ucla.edu/ungc). The GRCh37/hg19 reference genome was used to map reads. GATK was employed for joint-called variants [1], whereas Annovar and the Ensembl Variant Effect Predictor tool was used for annotation [2, 3].

**C9orf72 repeat screening**. The technique for screening repeats was fluorescent and repeat-primed PCR [4]. An ABI 3730 genetic analyzer allowed the analyses of fragment’s length. The Peak Scanner Software was also employed for data analysis.

**Screening of the main dementia genes.** The screening was applied for known (AD&FTD Mutation Database: http://www.molgen.ua.ac.be/ADMutations) or novel variants (classified according to the American College of Medical Genetics and Genomics and the Association for Molecular Pathology (ACMG-AMP) guidelines [5]). The analysis focused on both the coding and exon-intron region of the following genes: GRN, MAPT, TARDBP, FUS, and APP, PSEN1, PSEN2, using the following transcripts as references: NM_001136129 (APP), NM_001170634 (FUS), NM_002087 (GRN), NM_001123066 (MAPT), NM_000021 (PSEN1), NM_000447 (PSEN2) and NM_007375 (TARDBP). Afterwards, Sanger sequencing was employed to confirm the variants.

**Dementia risk alleles.** Based on the sequencing data, the following genotypes were obtained: TREM2 R47H (rs75932628), MAPT A152T (rs143624519), as well as APOE (rs429358 and rs7412), and 17q haplotype (rs1560310). All the genetic methods and analyses were developed by Ramos – Coppola Lab (University California Los Angeles).

**S2. Results. Comparisons of cognition, social cognition and neuropsychiatric symptoms between all patients with mutations (GR1 grouped) vs. GR2.**

**GR1 vs. GR2 *H1H2* and *H2H2* genotypes**

GR1 patients exhibited early disease onset (see Table 1), although no differences in disease duration were observed in comparison to risk *tau* haplotypes (t = -2.16, p < .05, zCC = -2.05). GR1 patients also exhibited more impaired executive functioning (t = -2.87, p< .05, zCC = -2.06) and ToM (t = -3.05, p< .05, zCC = -2.11) in comparison to risk *tau* haplotypes. Conversely, patients with risk *tau* haplotypes showed greater chronic total neuropsychiatric symptoms (t = -2.99, p < .05, zCC = -2.09), apathy (t = -2.79, p < .05, zCC = -2.03), disinhibition (t = -3.21, p < .05, zCC = -2.33), and disorganized behavior (t = -3.01, p < .05, zCC = -2.11) compared to GR2 patients. No other analyses reached significant differences (Tables 1-2 and Figure 1D).

**GR1 vs. GR2 *APOE* Variants (*ε2ε3, ε3ε4, ε4ε4*)**

GR1 patients exhibited early disease onset (t = -3.05, p< .05, zCC = -2.11) without differences in disease duration, poorer executive functioning measure (t = -3.19, p< .05, zCC = -2,12), and more impaired ToM (t = -2.99, p< .05, zCC = -2.09) compared to patients with *APOE* variants. Furthermore, GR1 patients exhibited more current neuropsychiatric symptoms than patients with *APOE* variants as shown by total FrSBe scores (t = -3.03, p < .05, zCC = -2.10), apathy (t = -2.99, p < .05, zCC = -2.09), disinhibition (t = -2.89, p < .05, zCC = -2.04), and disorganized behaviors (t = -3.89, p < .05, zCC = -2.19). In contrast, risk *APOE* variants showed more chronic apathy (t = -3.19, p < .05, zCC = -2.14) and chronic disorganized behavior (t = -3.02, p < .05, zCC = -2.10) than GR1 patients. No other analyses reached significance (Tables 1-2 and Figure 1E).

**S3. Results. Comparisons of brain atrophy between all patients with mutations of GR1 grouped vs. GR2.**

**GR1 vs GR2 *tau* risk haplotypes**

GR1 patients exhibited increased atrophy than *tau* risk haplotypes in the right frontal lobe, right insula, bilateral precuneus, right dorsolateral prefrontal cortex, and right inferior temporal gyrus. In contrast, risk *tau* haplotypes showed increased atrophy in the bilateral superior parietal lobe and the right hippocampus (Table 3 and Figure 2D).

**GR1 vs GR2 *APOE* risk variants**

GR1 patients presented increased atrophy than patients with risk *APOE* variants in the right anterior cingulated cortices, bilateral precuneus, right caudate nucleus, and right inferior temporal gyrus. Conversely, risk *APOE* variants had more atrophy in right superior parietal cortices, right medial temporal gyrus, and right hippocampus (Table 3 and Figure 2E).

**Supplementary figures**

**
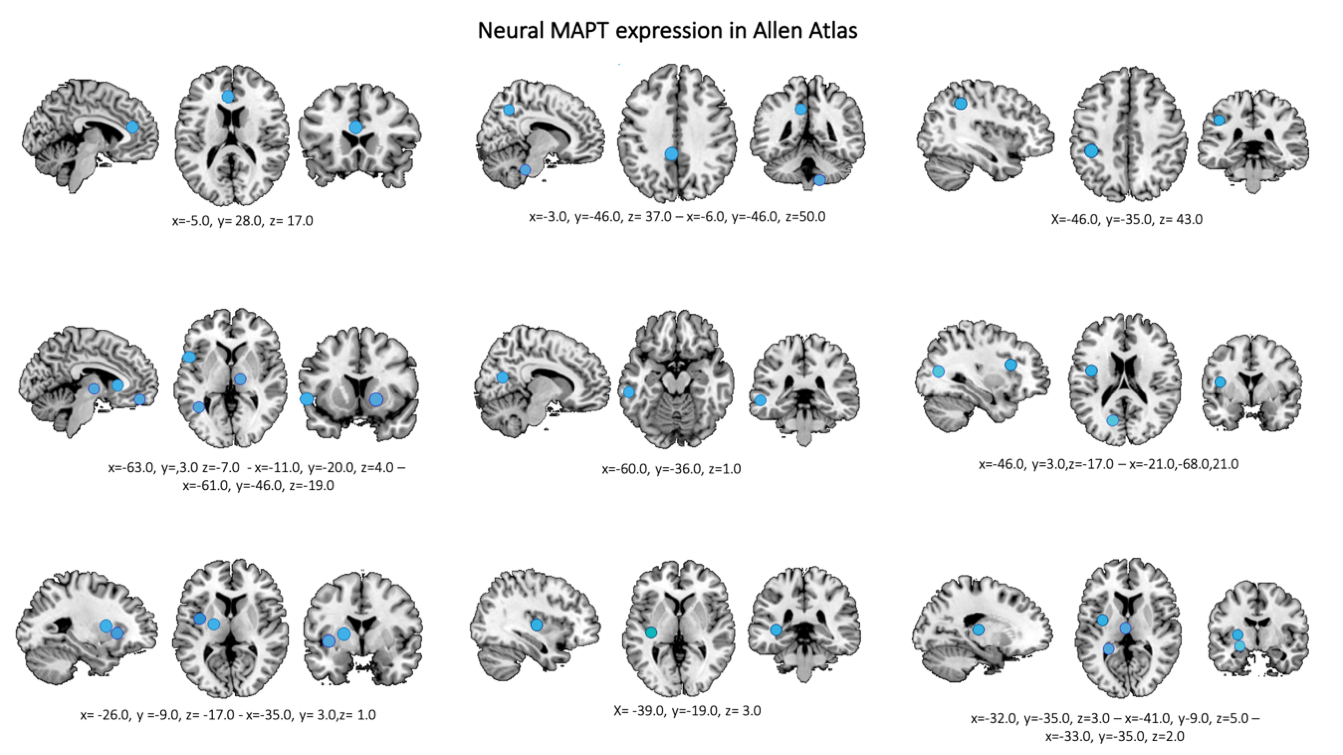
**

**Supplementary Figure 1.** Pattern of regions with high neural expression of MAPT gene, according to the Allen Human Brain Atlas (Allen Institute for Brain Science, [6, 7]).

**
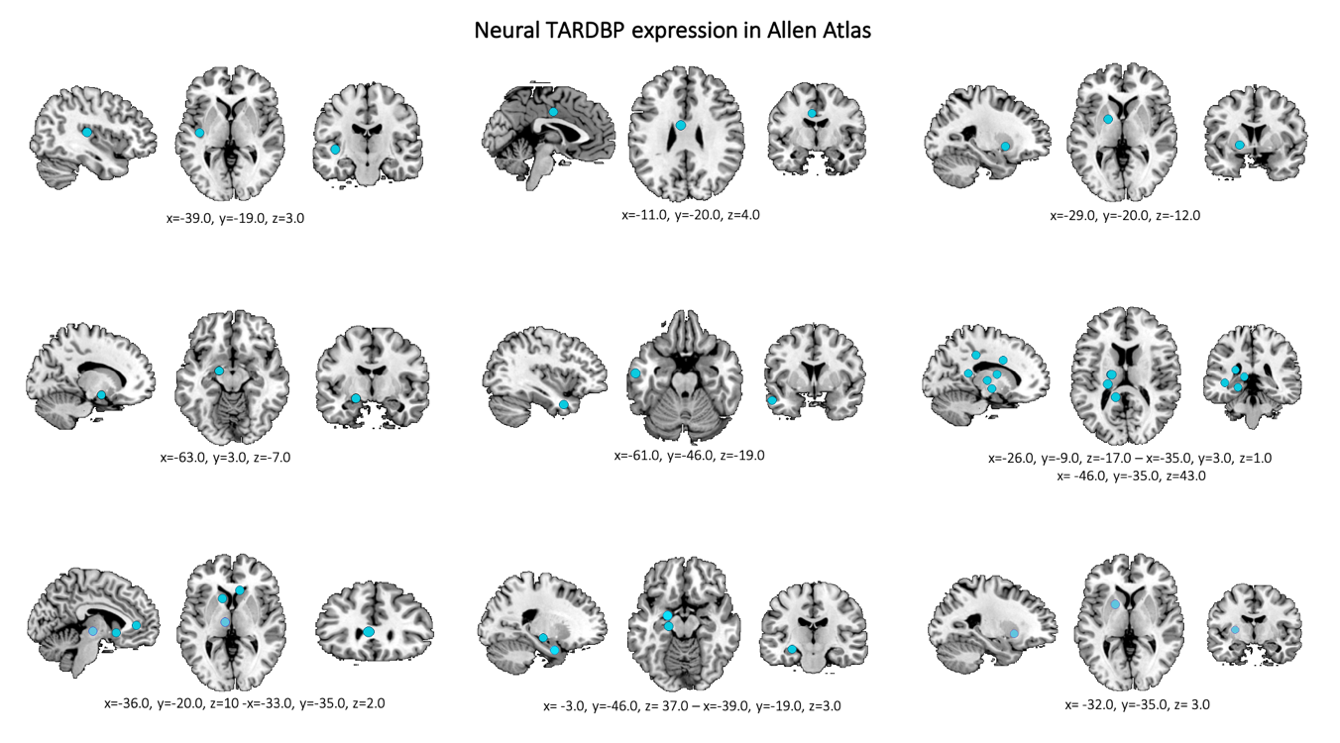
**

**Supplementary Figure 2.** Pattern of regions with high neural expression of TARDBP gene, according to the Allen Human Brain Atlas © (Allen Institute for Brain Science, [6, 7]).

**
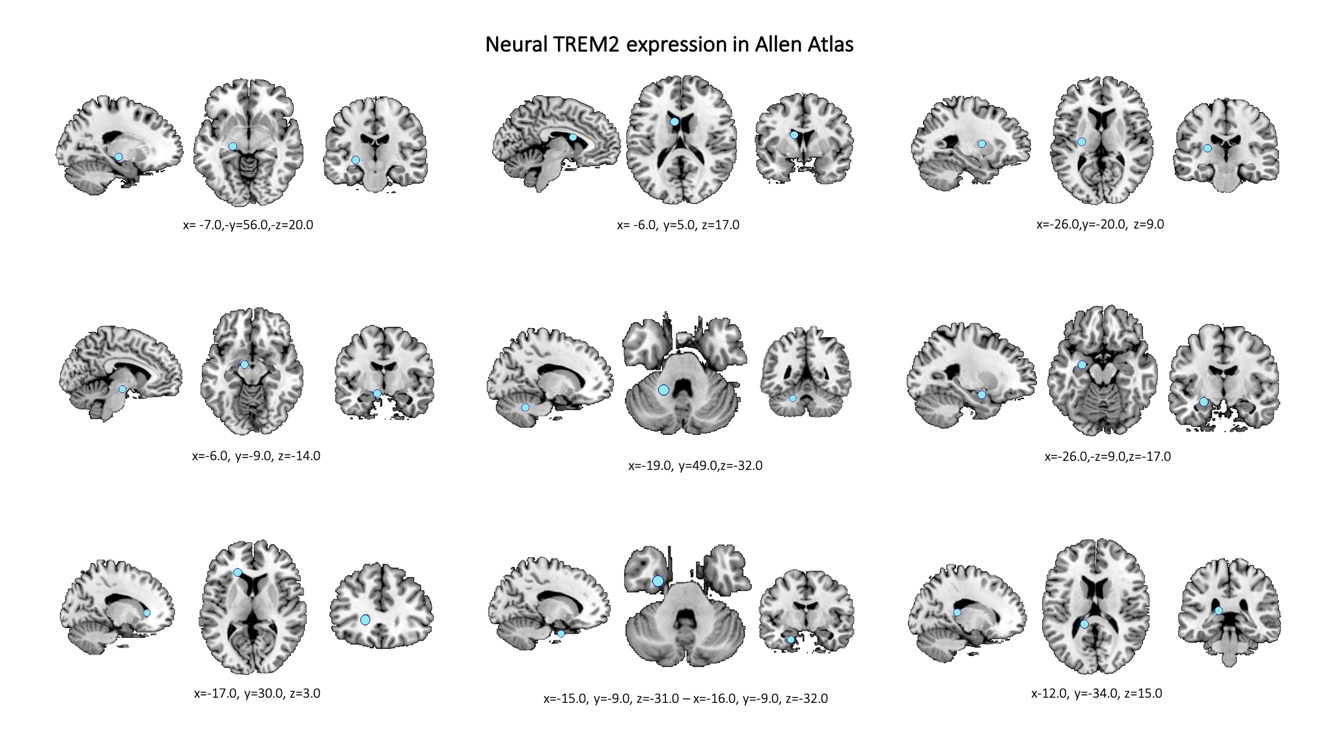
**

**Supplementary Figure 3.** Pattern of regions with high neural expression of TREM2 gene, according to the Allen Human Brain Atlas © (Allen Institute for Brain Science, [6, 7]).

**
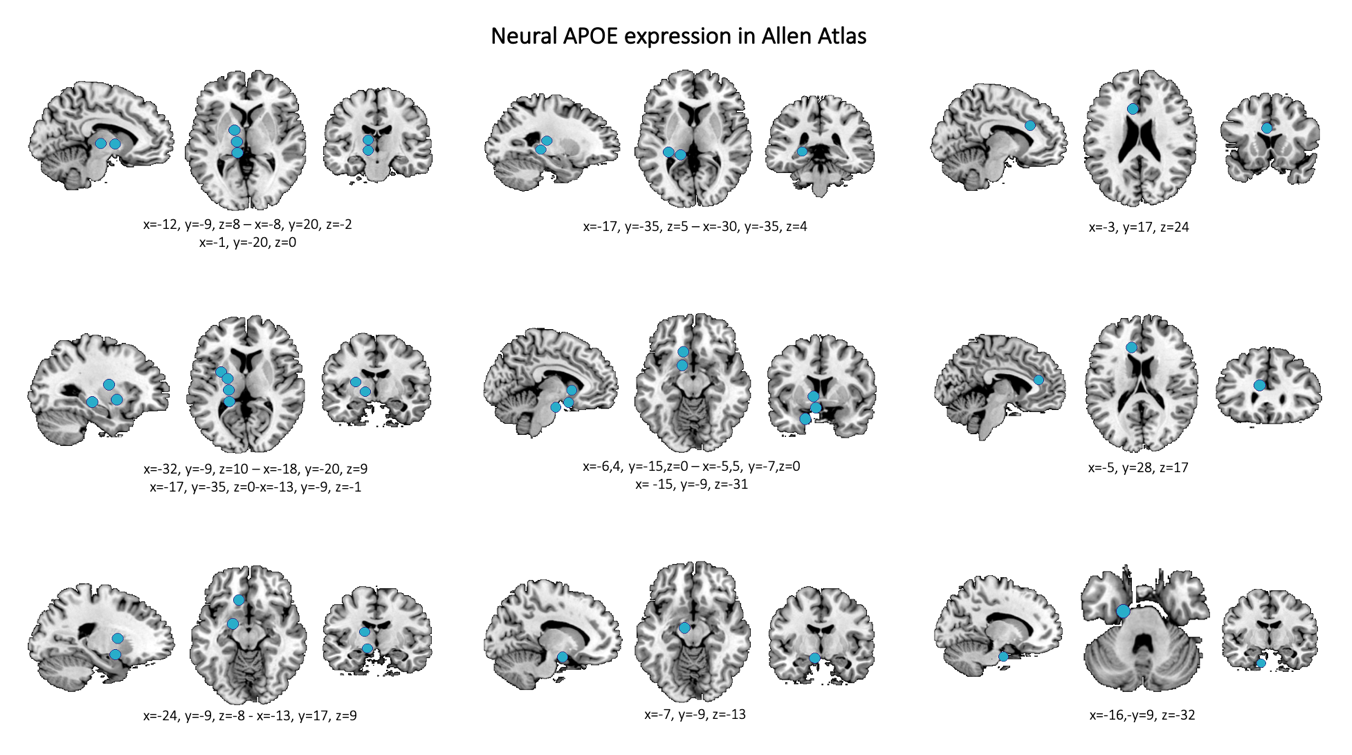
**

**Supplementary Figure 4.** Pattern of regions with high neural expression of APOE gene, according to the Allen Human Brain Atlas © (Allen Institute for Brain Science, [6, 7]).

**Supplementary References**

1. McKenna A, Hanna M, Banks E, Sivachenko A, Cibulskis K, Kernytsky A, et al. The Genome Analysis Toolkit: a MapReduce framework for analyzing next-generation DNA sequencing data. Genome research. 2010;20 9:1297-303.

2. McLaren W, Gil L, Hunt SE, Riat HS, Ritchie GRS, Thormann A, et al. The Ensembl Variant Effect Predictor. Genome Biology. 2016;17 1:122; doi: 10.1186/s13059-016-0974-4.

3. Wang K, Li M, Hakonarson H. ANNOVAR: functional annotation of genetic variants from high-throughput sequencing data. Nucleic Acids Res. 2010;38 16:e164; doi: 10.1093/nar/gkq603.

4. DeJesus-Hernandez M, Mackenzie IR, Boeve BF, Boxer AL, Baker M, Rutherford NJ, et al. Expanded GGGGCC hexanucleotide repeat in noncoding region of C9ORF72 causes chromosome 9p-linked FTD and ALS. Neuron. 2011;72 2:245-56.

5. Richards S, Aziz N, Bale S, Bick D, Das S, Gastier-Foster J, et al. Standards and guidelines for the interpretation of sequence variants: a joint consensus recommendation of the American College of Medical Genetics and Genomics and the Association for Molecular Pathology. Genetics in Medicine. 2015;17 5:405-23; doi: 10.1038/gim.2015.30.

6. Hawrylycz MJ, Lein ES, Guillozet-Bongaarts AL, Shen EH, Ng L, Miller JA, et al. An anatomically comprehensive atlas of the adult human brain transcriptome. Nature. 2012;489 7416:391-9.

7. Jones AR, Overly CC, Sunkin SM. The Allen Brain Atlas: 5 years and beyond. Nat Rev Neurosci. 2009;10 11:821-8; doi: 10.1038/nrn2722.
